# Emotional Intelligence and Knowledge Hiding Behaviors: The Mediating Role of Job Stress

**DOI:** 10.3389/fpsyg.2022.845782

**Published:** 2022-05-12

**Authors:** Xiangming Wang, Baobao Dong

**Affiliations:** School of Business and Management, Jilin University, Changchun, China

**Keywords:** emotional intelligence, job stress, knowledge hiding, knowledge management, cognitive appraisal theory of stress and coping

## Abstract

Emotion is fundamental to human experiences influencing our daily activities including cognition, communication, learning, and decision-making, but the effect of emotion on knowledge management in firms receives a little attention, especially in the field of knowledge hiding behaviors. Drawing on the cognitive appraisal theory of stress and coping as a unique theoretical lens to explicate how knowledge hiding behaviors happen, this study investigates the mediating effect of job stress in the relationship between emotional intelligence (EI) and knowledge hiding behaviors. We conducted a field study with 193 full-time employees in smart healthcare firms to test our hypotheses. Results supported the mediating effects of job stress in accounting for the relationship between EI and knowledge hiding behaviors. Our study is among the first to examine how emotional intelligence predicts knowledge hiding behaviors. This study contributes to the literature on knowledge management and emotional intelligence.

## Introduction

With the development of artificial intelligence (AI), big data, and clouding technologies, knowledge is becoming more and more important to the world economy, communities and firms. As the micro-foundation of the firm, knowledge management has gained increased attention in the past couple of decades (Loh et al., 2011; Zhang et al., 2021). The growth and market competitiveness of a firm largely rely on the knowledge management of its employees and teams (Koopman et al., 2020); the success of knowledge sharing also largely relies upon employees’ willingness and emotion in workplace (Shrivastava et al., 2021). But when facing fear or stress, employees are not voluntary to share their own knowledge or even hide the knowledge (Qureshi and Evans, 2015). Knowledge hiding behavior, defined as “a deliberate effort on the part of employees to hide or suppress important information that coworkers have asked for” (Syed et al., 2021, p. 5), has been found to be negative for efficiency and performance (e.g., Kyriacou, 2001; Ma et al., 2020). Despite such behavior’s negative connotation for firms, research needs to unveil the influencing factors to make this kind of behavior happen. One such antecedent that holds importance in the digital and AI era but that has received limited attention is emotional intelligence (hereinafter called EI), which refers to the ability of an individual to monitor his or her own feelings and emotions as well as those of others, to distinguish them, and to direct his or her thoughts and actions accordingly. EI is of help to harmonize relationships, lead to trust and is highly conducive to individual and organizational performance (Elfering et al., 2013).

Previous studies have already verified that employees’ decision to hide knowledge is influenced by various factors. For instance, research has emphasized job-related resources (Schieman, 2013), time pressure (Volkoff et al., 2010), leadership styles (Syrek et al., 2013), targets (Connelly and Zweig, 2015), and workplace ostracism (Zhao et al., 2016) to stop or promote knowledge hiding behaviors within organizations. Some researchers have called for the urgent need to unscramble the micro-foundation of knowledge hiding behaviors (DeCaro et al., 2011; Connelly et al., 2012; Feng and Wang, 2019). Given this, the role that EI can play in instigating knowledge hiding behaviors has been largely overlooked (Walter and Bruch, 2009; Troth et al., 2012). Recently, some studies have changed the status quo by investigating emotionally perceptive leaders (Vidyarthi et al., 2014) and emotion regulation (Walter et al., 2012; Tuncdogan et al., 2017) as crucial factors of employees` knowledge hiding behaviors. Nonetheless, the role that other emotions, such as EI’s, play in prompting knowledge hiding behaviors is an important unexplored field. *Although our comprehension of knowledge hiding behaviors has been increasingly dawned by scholars taking an cognitive appraisal perspective* (e.g., Peng, 2013; Vidyarthi et al., 2014)*, there is still an important gap regarding insights on how knowledge hiding behaviors can be traced to emotive factors, especially in the form of EI. In fact, EI can shape new understanding on current situation by exhibiting its specific functions, which can be an invisible hand to manipulate knowledge management behaviors that lead the firm forward. Thus, EI is critical to knowledge hiding behaviors. It goes without saying that it is a fearful appeal to analyze the influence of EI on knowledge hiding behaviors. To our knowledge, empirical research ls limited on this issue. In order to bridge this research gap, we empirically test the above relationship to provide a more insightful understanding of the possible impact of EI on knowledge hiding behaviors*.

Extant research also contends that EI, as a kind of abilities, cannot be directly transformed into “preventer” of knowledge hiding behaviors, there exists a certain mediation mechanism that can help EI realize “its dream” (Ohly and Fritz, 2010; Elfering et al., 2013). In order to answer recent calls, after considering the potential antecedents of knowledge hiding behaviors, we suggest job stress as an important underlying mechanism through which EI exhibits its favorable effects on employees’ knowledge hiding behaviors. The experience of job stress is the psychological state that represents an imbalance between employees’ perceptions of the demands placed on them and their ability to cope with those demands in workplace (Lindebaum and Jordan, 2014; Ma et al., 2020). By far the most frequently cited response to job stress is dissatisfaction with the job, which in turn can affect knowledge management behaviors (e.g., knowledge hiding and sharing). Therefore, this study aims to advance knowledge on employee-specific emotive factors that can influence knowledge hiding behaviors. Psychological ownership theory (Peng, 2013) and conservation of resources (Russell et al., 2009; Jahanzeb et al., 2019) argued the antecedents and consequences of employees’ knowledge hiding behaviors, while other theoretical perspectives of explaining employees’ knowledge hiding behaviors are ignored. Therefore, we use the cognitive appraisal theory of stress and coping as a theoretical lens to elaborate how EI illustrate their beneficial effects on avoiding employees’ knowledge hiding behaviors.

Our study makes several contributions to the EI and knowledge management literature by addressing the following key points. First, our study contributes to an interesting domain of knowledge hiding behaviors where we offer two particular antecedents (EI and job stress) of knowledge hiding behaviors (DeCaro et al., 2011; Tuncdogan et al., 2017). Second, our research shed lights on the emerging field of EI (Lindebaum and Jordan, 2014) by linking EI with employees’ knowledge hiding behaviors through the underlying job stress. Third, the present research responds to the calls by using special theoretical lens to unravel the mechanism of employees’ knowledge hiding behaviors.

## Theory and Hypotheses

### EI and Knowledge Hiding Behaviors

We employ the cognitive appraisal theory of stress and coping (Lazarus and Folkman, 1984) to elaborate on the mediating role of job stress in the EI–employees’ knowledge hiding behaviors relationship. According to this theory, a stressful situation and the resulting outcomes is a dynamic interaction between a person and his/her environment. This theory tells why some individuals manage to perform well in stressful situations while others fail to do so (Lazarus and Folkman, 1984). There are two processes primarily referred to as primary and secondary appraisal by evaluating the detrimental or beneficial environment (Smith and Lazarus, 1993). The primary appraisal implies that when individuals face a stressful situation, they cognitively appraise the situation as a challenge or hindrance. Once the individual has determined whether the stressful situation is causing him/her harm or benefit, then going into the secondary appraisal process. In this stage, the individual figures out how he/she will cope with the situation. Secondary appraisal along with an individual’s primary appraisal determines the outcomes generated (Griner and Smith, 2000). In line with this theory, we argue that whenever an individual becomes a victim of job stress, he/she might appraise or take the situation as a threat to restrict his or her behaviors. While facing EI, employees can find comfortable situation to express themselves. This in turn leads to less knowledge hiding behaviors.

Knowledge hiding behaviors refers to a deliberate effort on the part of employees to hide or suppress important information that coworkers have asked for (Connelly et al., 2012; Nguyen et al., 2022). There are three dimensions of knowledge hiding behaviors, and they are playing dumb, evasive hiding, and rationalized hiding (Connelly et al., 2012). If individual is unconscious of information need from others then playing dumb arises. If individual provides incorrect information on purpose or pretends to help other while in reality he/she does not have any intention to respond then evasive hiding occurs (Jha and Varkkey, 2018; Ghani et al., 2019). If an individual cannot provide the requested information due to its confidential nature or because the supervisor has not allowed him/her to share this information, then rationalized hiding appears. Previous studies showed that job-related resources (Schieman, 2013), time pressure (Volkoff et al., 2010; Shrivastava et al., 2021), leadership styles (Syrek et al., 2013), targets (Connelly and Zweig, 2015), and workplace ostracism (Zhao et al., 2016) influence knowledge hiding behaviors, but only limited attention has been paid to the role of emotion in influencing knowledge hiding behaviors, for example, EI.

Based on the Theory of Multiple Intelligence, EI, proposed by Salovey and Mayer (1990, p. 186) can be defined as “an ability to perceive and distinguish the emotions of oneself and others, and to use the information obtained as a basis for guiding one’s thoughts and actions.” In their study, they argued that EI includes three processes, and they are emotions assessment and expression, emotions adjustment, and emotions application. But Mayer and Salovey (1997) revised the definition of EI in view of the fact that most of the early studies of EI focused on emotional awareness and regulation, rather than thinking about feelings. They defined EI as the ability to perceive, express, and evaluate emotions; to aid in the production of thinking power through emotion; the ability to understand and improve emotional knowledge. Bar-On (2000) argued that EI is not only an ability that can affect a person’s success in dealing with various requirements of the environment and bearing pressure, but also requires non-cognitive skills and ability. Therefore, they conceptualized EI as consisting of four processes: self-emotion appraisal (SEA: The ability to gain insight into one’s own emotions and the ability to express your emotions naturally), others’ emotion appraisal (OEA: The ability to perceive and understand the emotions of others), use of emotion (UOE: The ability to regulate one’s emotions and recover quickly from sadness), and regulation of emotion (ROE: The use of personal emotion that motivates people to formulate actions and express themselves). EI integrates the four processes together to influence the thinking and behaviors of self and others (Troth et al., 2012). The former two processes focus on how much attention an individual is willing to pay to his or others` emotions and feelings, and whether he can accept the influence of such emotions. The latter two processes concentrate on whether an individual has the ability to improve negative emotions and channel them into positive ones or use positive emotions to influence others` behaviors. The four interrelated processes strengthen the structure of EI and should be integrated into one concept, which can implicitly clarify the connotation of EI. So even EI is divided into four processes, we still collapse EI to single factor.

Obviously, all emotional expression may influence employees` knowledge hiding behaviors. Job stress negatively affects safety behavior if employees cannot express their feeling effectively, thus reducing knowledge sharing (Lu and Kao, 2013) and enhancing hiding behaviors. In the view of trait competitiveness, EI may reduce knowledge hiding behaviors in workplace (Peng et al., 2020). Extant studies show that EI can improve personal psychological wellbeing, reduce stress and job dissatisfaction, and improve employee performance. MacCann et al. (2019) defined EI as an emotional trait derived from personality. EI also has a profound influence on individual work behaviors. EI is produced in the process of interaction with others. During the internal interaction in firms, if employees with high EI can carefully observe colleagues` negative emotion, and appropriately express the feeling of care, and ask colleagues to exert emotional expression, it is conducive to employees to express their knowledge ideas and avoid the occurrence of knowledge hiding behavior (Tugade and Fredrickson, 2007). In the same way, when managers can detect and distinguish the emotional changes of employees, and then analyze and adjust their own thinking and actions with these messages, thus improving employees` behavior of communicating knowledge with others rather than hiding knowledge. So we put forward the first hypothesis:

*Hypothesis* 1: EI is negatively associated with knowledge hiding behaviors.

### The Mediating Role of Job Stress

Work stress is a subjective and individual phenomenon (Ladegaard et al., 2019). It describes a person’s cognitive assessment of environmental events and his ability to adapt to them, so it cannot be described by objective stimuli (such as job requirements or environmental events) or some adaptation (a certain state or level of individual physical and mental health). If the individual is out of harmony with the work environment, resulting in the individual’s mental, physiological, and cognitive responses to lose balance, such a state is job stress. Li et al. (2019) argued that employees respond to stress based on their perception of a potential stressor rather than the stressor itself. Stress in the workplace has an impact not only on employees, but also on the future development of the organization. An imperfect work environment will not only cause negative effects, such as unhealthy physical and mental health of employees, lack of positive work motivation, and reduced work quality, but also cause the organization to pay extra costs for recruiting and training personnel due to the high turnover rate of employees (Loh et al., 2011). Therefore, job stress cannot be underestimated, which requires managers to pay attention to.

Netemeyer et al. (2005, p. 132) defined job stress as “the nervousness/anxiety associated with the job, affecting an employee’s emotional and/or physical health,” it is produced after the interaction between people and the working environment. All the stimulation caused by work factors and the emotional reaction of tension and anxiety can be called job stress. The job stress of employees refers to the experience that employees perceive unpleasant and negative emotions in the work field, including anger, anxiety, tension, frustration, or depression, etc. These negative emotional experiences are perceived by employees in the work situation and then affect their behaviors (Kyriacou, 2001).

Using the cognitive appraisal theory of stress and coping (Lazarus and Folkman, 1984), we extend this recent line of inquiry and suggest EI as one of the important interpersonal factors that influence employees’ decision to hide or show knowledge from others at work. Further, knowledge hiding behaviors are an important self-protective mechanism that employees use to cope because of repressive work environment that further influences their work-related attitudes and behaviors (Jha and Varkkey, 2018; Li et al., 2019).

For employees, the organization is also an important source of social and emotional resources. When employees believe that the organization is the source of injury and obstruction (Gibney et al., 2009) and the organization treats employees in a negative way, they can perceive job stress (Škerlavaj et al., 2018). At this point, employees will repay the organization in retaliation (Gibney et al., 2009). This kind of negative social exchange relationship (Gibney et al., 2009) will bring a series of consequences. For example, employees may respond to the organization by willful absenteeism, anti-organizational citizen behavior and other intentional behaviors that clearly violate the organization’s norms and legitimate interests (Qureshi and Evans, 2015), or deliberately hide knowledge (Zhang et al., 2021). Employees may also feel stressed if they have concerns about how the organization will handle criticism and dissent. Based on the standpoint of self-protection and reducing the risk of being treated improperly by the organization, employees may deliberately hide their knowledge in retaliation to the organization with the mentality of watching the show. Negative reciprocity can also bring job stress to employees (Gibney et al., 2009). If specific job stress makes employees feel uncomfortable, they will take the negative coping strategies to conceal knowledge or retain knowledge to improve their irreplaceable status (Hernaus et al., 2019), and increase their bargaining power with organizations in exchange for reducing their job stress or unfavorable situation (Lu and Kao, 2013). It can be seen that job stress affects employees’ knowledge hiding behavior.

As for the employees with higher EI, they have a lower feeling of working pressure and are more able to present a better working state (Xiong et al., 2021). Especially when facing difficulties from supervisors and colleagues, they are more able to turn pressure into motivation and reduce their own job stress. Vidyarthi et al. (2010) argued that EI can make people feel happy, relieve inner fear and anxiety, and make people feel less pressure. EI can affect the physical and mental development of individuals. If individuals can detect their own and others’ emotions, perceive emotions, learn to control their emotions, and then have a good interaction with others (Walter et al., 2012), they can feel the benefits brought by positive emotions and enjoy a harmonious working environment (Van Rooy and Viswesvaran, 2004). On the contrary, when individuals have negative emotions, they often show irrational behaviors, fail to make right choices, and even cause irreparable consequences. Therefore, better use and regulation of emotions can not only help individuals to change their negative emotions and adapt to the environment appropriately, so as to establish a positive and optimistic emotion (Laurijssen and Glorieux, 2013), but also empathize with others’ emotions and communicate with colleagues peacefully, so as to have a good working relationship and reduce job stress (Kühnel et al., 2012; Wressle and Samuelsson, 2014). Therefore, it can be seen from the above that emotion appraisal, emotion expression, emotion use, and emotion regulation are of great importance to relieve job stress.

Because EI are more likely to take effect by appraising self and others emotion and regulate emotions effectively so that employees’ self-interests are met, such behaviors can reduce job stress, which in turn can make them less use coping strategies, such as hiding knowledge. When employees perceive the nervousness/anxiety associated with the job, they may purposefully hold back information from coworkers by saying that it is confidential (rationalized hiding) or promising that they will share it but later back off from this promise (evasive hiding). Therefore, we hypothesize the following:

*Hypothesis* 2: Job stress has a positive effect on employees` knowledge hiding behaviors.

*Hypothesis* 3: Job stress mediates the relationship between EI and employees` knowledge hiding behaviors.

**Figure 1:**
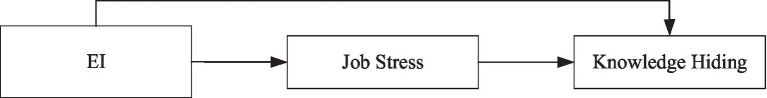
Theoretical model.

Figure 1 depicts our conceptual model.

## Materials and Methods

### Sample and Data Collection

Full-time employees at 10 listed companies in smart healthcare industry located in Beijing–Tianjin region and Yangtze River Delta in China were invited randomly to participate in our study because internal knowledge exchange is frequent in high-tech companies like that in Smart Healthcare industry (Walter et al., 2012). One of the authors contacted the human resources department of the firms to introduce the research project and asked the director of HR for survey help. The 10 firms agreed to participate in the survey. We assured of the confidentiality of their responses to all participants. We made the survey face to face to ensure data quality.

We used a three-wave research design, which allowed us to temporally segregate the measurement of our predictor (T1: EI), mediator (T2: job stress), and outcome variables (T3: knowledge hiding behaviors). The time lag between each measurement point was 1 month. Adopting a temporally segregated design helps reduce potential concerns arising from solely using self-reported and single-source data collection methods (Tuncdogan et al., 2017). The surveys were collected during employees’ lunch time.

The data collection process lasted 3 months. We distributed 494 questionnaires out of which 271 of these were completed at T1; then, 246 respondents (25 participants at T1 refused to participate at T2) answered the surveys at T2, and 241 surveys were useful for the research, and 226 surveys (seven quit the job and eight refused to participate at T3) are collected at T3. After deleting invalid data with missing key items or selecting multiple consecutive items consistently, we got 193 usable data with a valid rate of 39.07%. The majority of the participants were male (67.36%), with an average age of 34.57 years (SD = 3.89) and average tenure of 4.34 years (SD = 2.58).

### Measurement

Because the measurement items of the variables in this study were originally developed in English. We strictly followed the back-translation procedures proposed by Brislin (1970) and translated these items into Chinese. We use a five-point Likert-type scale to evaluate the items (1 = strongly disagree, 5 = strongly agree). All the measurement items we used in our study are shown in Appendix.

#### Knowledge Hiding

Connelly et al. (2012) argued that knowledge hiding behavior, in real workplace, may include many examples. For example, one employee may ask a coworker for a copy of a report; the coworker may then reply that this report is confidential and that she will therefore not disclose it (rationalized hiding). In this example, the requested knowledge is not forthcoming, even though no deception is involved. Another example of knowledge hiding would be a situation in which the coworker provides some, but not all, of the requested knowledge (evasive hiding); in this case, deception may be involved. That is, hiding is not always deceptive; similarly, managers do not view hiding knowledge as deception (Takala and Urpilainen, 1999). Furthermore, knowledge hiding may have positive intentions or outcomes; as with any “white lie” (Saxe, 1991), it may be intended to protect the other party’s feelings, preserve confidentiality, or protect the interests of a third party. As such, it is necessary to evaluate knowledge hiding behavior in three ways: rationalized hiding, evasive hiding, and playing dumb. Besides, Syed et al. (2021) also believed that knowledge hiding behavior may contain two or more hiding behavior, such as “agreed to help him/her but never really intended to give help” and “said that I did not know, even though I did.” The measures of knowledge hiding behavior with three dimensions have been validated by Syed et al. (2021) and Shrivastava et al. (2021). Therefore, we use the operational definition of Connelly et al. (2012) to measure knowledge hiding behavior and integrate three dimensions into one single factor. Knowledge hiding behaviors is measured by a 12-item scale. It contains three dimensions: playing dumb (with Cronbach’s alpha = 0.79), evasive hiding (with Cronbach’s alpha = 0.86), and rationalized hiding (with Cronbach’s alpha = 0.82). Cronbach’s alpha reliability of this scale in the current study is 0.84.

#### Job Stress

We measured job stress using Netemeyer et al. (2005) four-item scale. Cronbach’s alpha for this measure was 0.78.

#### Emotional Intelligence

As we have elaborated above, EI includes four sequential processes: self-emotion appraisal, others’ emotion appraisal, use of emotion, and regulation of emotion. But these four processes are sequential and correlated and they cannot be divided into different dimensions. We use Wong and Law (2002) sixteen-item scale to measure EI because these items can express one’s own emotion and appraise others` emotions, then use the emotions to scan the feelings to reappraise or suppress emotions to realize the emotive purpose (Wong and Law, 2002; Van Rooy and Viswesvaran, 2004). All of these are crucial for the understanding of the concept of EI. Therefore, we use operational definition of EI of Wong and Law (2002), which is an independent variable in psychological research domain.

Cronbach’s alpha for this measure was 0.74. Self-emotion appraisal was measured using four-item scale of Wong and Law (2002). Cronbach’s alpha was 0.79. Others’ emotion appraisal was measured using four-item scale of Wong and Law (2002). Cronbach’s alpha was 0.75. Use of emotion was measured using four-item scale of Wong and Law (2002). Cronbach’s alpha was 0.83. Regulation of emotion was measured using four-item scale of Wong and Law (2002). Cronbach’s alpha was 0.80.

#### Control Variables

Following the recommendation of previous studies (e.g., Chen et al., 2020), we controlled for employees’ gender (one for male and zero for female), age, and tenure (period in current firm), because these demographic characteristics are likely to influence participants’ emotion and engagement in workplace (Benefiel, 2005). Prior research suggested that education level (1 = bachelors and below, 2 = masters and above; Sonnentag et al., 2014) and experience (0 = having no experience in your industry before working with current firms while 1 = having experience in your industry before working with current firms; Aránega et al., 2020) is an essential factor facilitating knowledge management behaviors.

### Common Method Bias

Common method bias refers to the bias that is caused by measurement error than to the variable of the study (Bagozzi and Yi, 1991). Although our data were collected at different period of time, all variables were collected from the same source, thus causing common method biases. In order to check if common method variance can be a big concern in this study, following the recommendation of Podsakoff et al. (2003), Harman’s one-factor test was conducted with the principal axis factoring method and constrained the analysis to no rotation to make sure whether the first variable can explain most of the variance, and we found that the first factor can only explain 37.33% of variance. Then we included the 32 items collected from the same employee into one model and compared its model fit indices with the measurement model. We found that the one-factor model had a poor fit with the data set (*χ*^2^ = 596.31, df = 138, *χ*^2^/df = 4.32, CFI = 0.76, NFI = 0.79, and RMSEA = 0.15). Hence, common method bias cannot be a concern in this study.

### Confirmatory Factor Analysis

To evaluate the construct validity of the key variables, we conducted confirmatory factor analyses (CFA) in Table 1. Following Anderson and Gerbing (1988), we conducted one-on-one CFA by pairing a two-factor model with a single-factor model. The CFA findings indicated a three-factor model has better results of goodness of fit indices (*χ*^2^ = 244.91, df = 119, *χ*^2^/df = 2.06, CFI = 0.98, NFI = 0.96, and RMSEA = 0.06) as shown in Table 1, as compared to other models, which proved the distinctiveness of the hypothesized model used in the present study. So we confirmed that the hypothesized four-factor model fit the data better than all the alternative models.

**Table 1:** Results of confirmatory factor analyses (CFA; *N* = 193).

Variable	*χ* ^2^	df	CFI	NFI	RMSEA
Hypothesized model (three-factor model)	244.91	119	0.98	0.96	0.06
Two-factor model (EI and job stress combined into one factor)	363.08	124	0.89	0.86	0.10
One-factor model	596.31	138	0.76	0.79	0.15

## Results

Table 2 presents the correlation matrix of the variables. We can see from Table 2 that knowledge hiding behaviors is significantly related to EI (*r* = −0.23, *p* < 0.01) and job stress (*r* = 0.30, *p* < 0.001), respectively.

**Table 2:** Correlation matrix (*N* = 193).

Variables	1	2	3	4	5	6	7	8
1. Age	1							
2. Gender	−0.04	1						
3. Tenure	−0.01	0.05	1					
4. Education level	−0.06	−0.03	0.10	1				
5. Experience	−0.06	0.03	−0.09	0.04	1			
6. EI	0.02	−0.09	−0.10	0.07	0.24^**^	1		
7. Job stress	−0.01	−0.07	0.06	0.10	−0.19^*^	−0.37^***^	1	
8. Knowledge hiding	−0.02	−0.09	0.12	0.12	0.07	−0.23^**^	0.30^***^	1
Mean	34.57	0.76	4.34	1.67	0.67	3.93	3.88	302
S.D.	3.89	0.18	2.58	0.83	0.21	0.84	0.67	0.72

* *p* *< 0.05;*

** *p* *< 0.01;*

*** *p < 0.001*.

After evaluating the factor construct and common method bias, we then test all hypotheses by using hierarchical linear regression as shown in Table 3. As predicted in hypothesis 1, there is a negative relationship between EI and knowledge hiding behaviors. The results of in model 2 of Table 3 show that EI has a significant negative impact on knowledge hiding behaviors (*β* = −0.37, *p* < 0.01), thus supporting hypothesis 1. As predicted in hypothesis 2, there is a positive relationship between job stress and knowledge hiding behaviors. The results in model 3 show that job stress has a significant positive impact on knowledge hiding behaviors (*β* = 0.23, *p* < 0.01), which offer support for hypothesis 2. In model 4, following procedures of testing mediating effect of Baron and Kenndy (1986), we put EI and job stress in the regression model. After combining the results of models 2 and 3, we found that EI has no significant effect on knowledge hiding behaviors and the coefficient of job stress on knowledge hiding behaviors is 0.19 (*p* < 0.01), less than its independent effect on knowledge hiding behaviors, thus supporting hypothesis 3, that is, job stress plays a full mediating role between EI and knowledge hiding behaviors.

**Table 3:** Results of hierarchical linear regression.

	Model 1	Model 2	Model 3	Model 4
**Controls**				
Age	−0.07 (0.19)	−0.08 (0.20)	−0.10 (0.22)	−0.09 (0.20)
Gender	−0.02 (0.36)	−0.02 (0.37)	−0.06 (0.37)	−0.08 (0.39)
Tenure	0.10 (0.51)	0.09 (0.50)	0.11 (0.51)	0.14 (0.53)
Education	0.10 (0.44)	0.11 (0.45)	0.09 (0.46)	0.10 (0.45)
Experience	0.14 (0.33)	0.16 (0.34)	0.18 (0.33)	0.15 (0.35)
**Independent variable**				
EI (H1)		−0.37^**^ (0.14)		−0.19 (0.16)
**Mediator**				
Job stress (H2)			0.23^*^ (0.11)	0.19^*^ (0.10)
*R* ^2^	0.16	0.20	0.28	0.30
Adjusted *R*^2^	0.13	0.17	0.25	0.26
*△R* ^2^	--	0.04	0.08	0.02
*F*-value	9.73^***^	11.85^***^	14.94^***^	18.79^***^
*N*	193	193	193	193

* *p* *< 0.05;*

** *p* *< 0.01;*

*** *p < 0.001*.

## Discussion

Our study is among the pioneer to examine the effects of EI on knowledge hiding behaviors in workplace in high-tech industry. Based on the cognitive appraisal theory of stress and coping (Lazarus and Folkman, 1984), this study demonstrated the influencing mechanism of EI on knowledge hiding behaviors. Specifically, our findings indicate that EI, as a kind of ability of emotion regulation, is one of vital antecedents of knowledge hiding behaviors. Furthermore, job stress were found to serve as the mediating factor that account for the impact of EI on knowledge hiding behaviors. The above results show that EI can influence individual feeling and efficacy in workplace through causal reasoning. When employees feel, control, or even appraise emotions and make correct attributions to past events or negative affairs, they can regulate their emotions, thus reducing their stress and fear in jobs, which is conducive to avoid knowledge hiding behaviors. However, employees with low EI may not adjust their emotions to their jobs (Ladegaard et al., 2019) or even cope with others` negative emotions in workplace, thus reducing their sense of efficacy and causing knowledge hiding behaviors. Our findings have important theoretical and managerial implications.

### Theoretical Contributions

Our study offers several theoretical contributions that extend the knowledge management and EI literature. First, our research uses the cognitive appraisal theory of stress and coping to examine the role of EI in impeding knowledge hiding behaviors. Although prior studies have found that EI is significantly related to job performance (e.g., Afolabi et al., 2010), problem-solving tactics (Li et al., 2019) and interpersonal relationships (Durland and Thomas, 2019) at employee level and firm growth and better performance at organizational level (e.g., Ma et al., 2020), its effect on knowledge hiding behaviors gained little attention. Our findings demonstrate that EI is essential for holding back knowledge hiding behaviors in workplace, which can be regarded as a kind of emotive relevance. Future research can take a cognitive appraisal view to find other valuable emotive factors to prevent the knowledge hiding behaviors.

Second, while most extant studies on EI rely upon emotion and ability theories (Hofmans et al., 2015; Shockley and Allen, 2015) to explain the impact of EI on employee behavior, we have used the cognitive appraisal theory of stress and coping to examine the effects of EI on knowledge hiding behaviors. Specifically, the mediating mechanism of job stress can explain the impact of EI on knowledge hiding behaviors from the view of cognitive appraisal, which can enhance our understanding of the role of EI in organizations.

Third, our results indicate that job stress plays a full mediating role between EI and knowledge hiding behaviors. This result further reinforces the long-held view of job stress as an emotive style because it can exert pressure and fear to employees, which can strengthen the positive impact on knowledge hiding behaviors (Shockley and Allen, 2015). In short, our findings to a large extent support the effectiveness of the path influence carried out by EI in shaping followers’ emotion and behavior.

Our study was conducted in China that owns the features of emerging and transitional economy. This offers a now-or-never chance to test the concepts developed in Western countries in an eastern culture. Specifically, the present study adds insights by interpreting novel mechanisms of EI in an eastern country context. China scores moderately high on power distance, collectivism, and *guanxi* with volatility, uncertainty, complexity, and ambiguity (VUCA; Siu et al., 2002). Past research has corroborated that EI is more likely to prevail in eastern cultures marred with the above cultural traits. China rates high on VUCA, which means that employees facing fear, risk, and ambiguity may take short-sighted measures to hide their knowledge without considering the damage imposed by these behaviors.

### Practical Implications

Our study provides valuable insights into how EI might promote employees to share their knowledge with coworkers but not hide knowledge even under job stress. First, our study shows that EI play an important role in facilitating the appraisal, use, and regulation of emotion for better knowledge management behavior. To avoid knowledge hiding behaviors at workplace, managers may show a strong EI to keep employees together as a driving force. Employees with high EI can cooperate the relationship with coworkers to set up harmonious atmosphere to facilitate cooperation. Besides, managers can encourage employees to face challenging goals to reduce the influence of job stress. Ambition sometimes can be of help for employees to regulate their emotion and avoid knowledge hiding behaviors. Managers must ensure open communication and close interpersonal interaction with their employees so that they feel safe to share knowledge with them. Mostly important, managers can build confidence in organizational vision and educate employees regarding the short and long-term goals and benefits of sharing knowledge with coworkers in the organization, which can hold back knowledge hiding behavior.

### Limitations of Future Directions

Though the current study puts forward a new perspective of EI, it is not free from limitations.

First, although our study used a time-lagged research design with data collected from independent sources, it cannot be classified as a pure longitudinal design as all the study variables were not tapped at all periods, which may cause complicated causal relationship among EI and knowledge hiding behaviors. Future researchers can use a longitudinal research design where all the research model variables are measured at all periods and check whether the results are general, then the relationships can be elaborated clearly in depth.

Second, generalization of the results is limited because the data comes from two most developed economic zone without considering the other underdeveloped and undeveloped areas. Therefore, researchers should make surveys in other zones, developed areas, such as the Pearl River delta, and underdeveloped, such as the northeast and the west areas. The comparison of the results from different areas can show us some insights in the near future.

Third, as we have elaborated above, EI and knowledge hiding behaviors have several processes or dimensions. Even we collapsed them into one single variables, it may be interesting to analyze the effects of different processes of EI on the different dimensions of knowledge hiding behaviors, for example, future research can focus on the influence of OEA on playing dumb, evasive hiding and rationalized hiding, thus inducing extraordinary results.

In all, in this study, we seek to extend extant knowledge on how EI influences knowledge hiding behaviors through workplace situation, namely, job stress from cognitive appraisal theory of stress and coping (Lazarus and Folkman, 1984). Job stress, on one hand, can power motivation for employees, and on the other hand, may have a baleful influence on the knowledge hider’s psychological state and even behaviors. Organizations should put more efforts to prevent or reduce the negative effects of such behavior through EI to promote effective knowledge management practices and harmonious knowledge sharing atmosphere.

## Data Availability Statement

The raw data supporting the conclusions of this article will be made available by the authors, without undue reservation.

## Author Contributions

XW designed the research model, collected the data, and wrote the sections "Materials and Methods," "Results," and "Discussion" of the article. BD wrote the other sections and processed the data. All authors contributed to the article and approved the submitted version.

## Funding

This research is financially supported from the National Natural Science Foundation of China under the grant no. 72072068.

## Conflict of Interest

The authors declare that the research was conducted in the absence of any commercial or financial relationships that could be construed as a potential conflict of interest.

## Publisher’s Note

All claims expressed in this article are solely those of the authors and do not necessarily represent those of their affiliated organizations, or those of the publisher, the editors and the reviewers. Any product that may be evaluated in this article, or claim that may be made by its manufacturer, is not guaranteed or endorsed by the publisher.

## Appendix

A measurement items of variables

Emotional intelligence (EI) (Wong and Law, 2002)

Self-emotion appraisal (SEA)

1. I have a good sense of why I have certain feelings most of the time.

2. I have good understanding of my own emotions.

3. I really understand what I feel.

4. I always know whether or not I am happy.

Others’ emotion appraisal (OEA)

5. I always know my friends’ emotions from their behavior.

6. I am a good observer of others’ emotions.

7. I am sensitive to the feelings and emotions of others.

8. I have good understanding of the emotions of people around me.

Use of emotion (UOE)

9. I always set goals for myself and then try my best to achieve them.

10. I always tell myself I am a competent person.

11. I am a self-motivated person.

12. I would always encourage myself to try my best.

Regulation of emotion (ROE)

13. I am able to control my temper and handle difficulties rationally.

14. I am quite capable of controlling my own emotions.

15. I can always calm down quickly when I am very angry.

16. I have good control of my own emotions.

Job Stress (Netemeyer et al., 2005)

1. My job tends to directly affect my health.

2. At the end of the day, my job leaves me “stressed-out.”

3. Problems associated with work have kept me awake at night.

4. I feel fidgety or nervous because of my job.

Knowledge hiding behaviors (Connelly et al., 2012)

Playing dumb

1. Pretended that I did not know the information.

2. Said that I did not know, even though I did.

3. Pretended I did not know what s/he was talking about.

4. Said that I was not very knowledgeable about the topic.

Evasive hiding

5. Agreed to help him/her but never really intended to.

6. Agreed to help him/her but instead gave him/her information different from what s/he wanted.

7. Told him/her that I would help him/her out later but stalled as much as possible.

8. Offered him/her some other information instead of what he/she really wanted.

Rationalized hiding

9. Explained that I would like to tell him/her, but was not supposed to.

10. Explained that the information is confidential and only available to people on a particular project.

11. Told him/her that my boss would not let anyone share this knowledge.

12. Said that I would not answer his/her questions.
